# ABBV-744 as a potential inhibitor of SARS-CoV-2 main protease enzyme against COVID-19

**DOI:** 10.1038/s41598-020-79918-3

**Published:** 2021-01-08

**Authors:** Zeynab Fakhar, Shama Khan, Suliman Y. AlOmar, Afrah Alkhuriji, Aijaz Ahmad

**Affiliations:** 1 Molecular Sciences Institute, School of Chemistry, University of the Witwatersrand, PO WITS, Johannesburg, 2050 South Africa; 2 Department of Clinical Microbiology and Infectious Diseases, School of Pathology, Faculty of Health Sciences, University of the Witwatersrand, Johannesburg, 2193 South Africa; 3 Department of Zoology, College of Science, King Saud University, P.O. Box 2455, Riyadh, 11451 Saudi Arabia; 4 Infection Control, Charlotte Maxeke Johannesburg Academic Hospital, National Health Laboratory Service, Johannesburg, 2193 South Africa

**Keywords:** Computational biology and bioinformatics, Drug discovery, Chemical biology, Computational chemistry

## Abstract

A new pathogen severe acute respiratory syndrome coronavirus 2 (SARS-CoV-2) has spread worldwide and become pandemic with thousands new deaths and infected cases globally. To address coronavirus disease (COVID-19), currently no effective drug or vaccine is available. This necessity motivated us to explore potential lead compounds by considering drug repurposing approach targeting main protease (M^pro^) enzyme of SARS-CoV-2. This enzyme considered to be an attractive drug target as it contributes significantly in mediating viral replication and transcription. Herein, comprehensive computational investigations were performed to identify potential inhibitors of SARS-CoV-2 M^pro^ enzyme. The structure-based pharmacophore modeling was developed based on the co-crystallized structure of the enzyme with its biological active inhibitor. The generated hypotheses were applied for virtual screening based PhaseScore. Docking based virtual screening workflow was used to generate hit compounds using HTVS, SP and XP based Glide GScore. The pharmacological and physicochemical properties of the selected lead compounds were characterized using ADMET. Molecular dynamics simulations were performed to explore the binding affinities of the considered lead compounds. Binding energies revealed that compound ABBV-744 binds to the M^pro^ with strong affinity (Δ*G*_bind_ −45.43 kcal/mol), and the complex is more stable in comparison with other protein–ligand complexes. Our study classified three best compounds which could be considered as promising inhibitors against main protease SARS-CoV-2 virus.

## Introduction

Coronavirus disease 2019 (COVID-2019) outbreak is a global pandemic caused by severe acute respiratory syndrome coronavirus 2 (SARS-CoV-2) which initially diagnosed in Chinese patients of Hubei’s Wuhan city in early December 2019^1^. SARS-CoV-2 disclosed a close genetic resemblance to the severe acute respiratory syndrome coronavirus (SARS-CoV) that already triggered an epidemic in 2003^2^. COVID-19 has been declared a global health disaster by World Health Organization (WHO) on 30 January 2020 as the disease hastily transmitted human-to-human and affected more than 170 countries across the world^3^. The existing condition is extremely increasing; therefore, the overall asperity of this disease persist to be serious. The infection rate of SARS-CoV-2 is higher (10–12%) in comparison with its mortality rate (5.4%)^4^. The most distinctive indications of COVID-19 patients are high fever, cough and excessive respiratory sickness that required urgent intensive care facility. Currently, there is no applicable and precise medication for the treatment of COVID-19, however, many drugs and vaccines are under clinical trials. The only practical approach available is the repurposing of existing antiviral drugs as these drugs have already been tested for their toxicity^5^. Still there is a prompt requirement to make substantial efforts to advance therapeutic interventions against CoV infections.

CoVs are single-stranded positive-sense RNA viruses belongs to the family of Coronaviridae. These viruses can be categorized into four species: alpha, beta, gamma and delta. The recent SARS-CoV-2 is from beta genus and is usually identified to affect commonly humans^6^. The RNA genome length of this virus is about 27–32 Kb encoding both structural and non-structural proteins. Among them, the structural proteins, membrane (M), envelope (E), nucleocapsid (N), hemagglutinin-esterase (HE) and spike (S) proteins contribute notably in viral transmission and replication in the host cells^7^. The 3C-like protease (3CLpro) protease plays critical role in the SARS-CoV-2 life cycle through virus replication and transcription process, thus studied as potential drug targets. Providing informative knowledge regarding the enzyme inhibition will be valuable in tailoring effective and selective inhibitors of 3CLpro as this enzyme is imperative for virus assembly and reproduction^8^.

**Figure 1:**
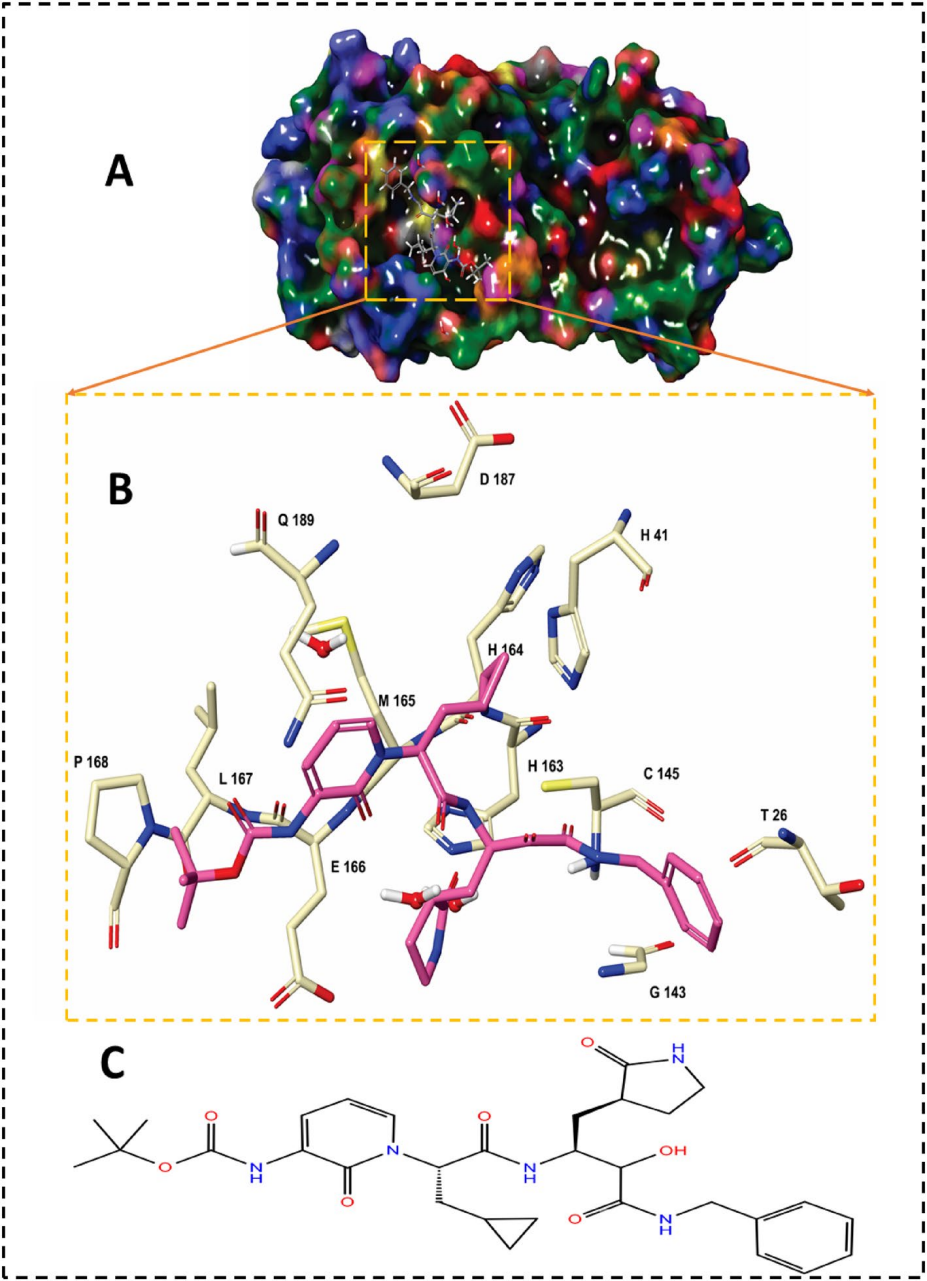
(**A**) Structural overview of M^pro^ enzyme in complex with α-ketoamide inhibitor (αk-13b) (magenta color); (**B)** Close up view of the binding pocket of M^pro^ accommodated αk-13b; **C**: 2D-chemical structure of α-ketoamide inhibitor (αk-13b).

The main protease (M^pro^) is a quintessential enzyme which contributes significantly in the life cycle of SARS-CoV-2 and inhibition of M^pro^ enzyme activity would block viral replication. Since no human proteases with a similar specified cleavage are characterized, thus the potential inhibitors are unlikely to be toxic. The M^pro^ enzyme consists of an asymmetric unit including 305 amino acid residues with CYS145 and HIS41 catalytic dyad in the active site^9,10^ (Fig. 1).

Experimental observations by Zhang et al*.*^10^ demonstrated the half maximal inhibitory concentration (IC50) value 0.67 µM for an α-ketoamide inhibitor (αk-13b) as a potent antiviral inhibitor against M^pro^ enzyme. Zhang et al*.* proposed the inhibition mechanism through a nucleophilic attack of the catalytic CYS145 to the α-keto group of the inhibitor and thiohemiketal moiety formation. This thiohemiketal group is stabilized by formation of several hydrogen bonds with the active residues of HIS41, GLY143, SER144 and CYS145, Fig. 1. This experimental observation proposed that α-ketoamide inhibitors interacted with the catalytic center of proteases through two hydrogen bond interactions whereas aldehydes and Michael acceptors only formed one hydrogen bond into the catalytic center of the target proteases^11,12^.

According to the aforementioned experimental information, we have chosen SARS-CoV-2 M^pro^ as a target enzyme to accelerate the prompt hunt of antiviral drug repurposing with the potential of gaining an effective short‐term solution to treat COVID‐19 patients.

To address this challenge, an specific library of anti and pro-viral agents including FDA approved drugs, compounds in clinical trials and preclinical compounds having inhibitory activity between 10 and 100 nM range against SARS-CoV-2 was considered for drug repurposing to attain immediate and precise results^13^. In this study, we developed an integrated approach of drug discovery integrating 3D structure-based pharmacophore modeling, virtual screening of 75 compounds library, molecular docking workflow, ADMET pharmacological analysis and molecular dynamics (MD) simulations. This scheme will provide an informative insight into the exploration of potent antiviral drugs, which could help in progressive attempts in the therapeutics of COVID-19.

## Methodology

### System preparation

The 1.95 Å crystal structure of SARS-CoV-2 main protease (M^pro^) in complex with α-ketoamide (αk-13b) inhibitor was extracted from the Protein Data Bank (PDB ID: 6Y2F)^10^. The structure of the enzyme was pre-processed, minimized and refined using the Protein Preparation Wizard^14^ implemented in Schrödinger suite. This involved eliminating of crystallographic waters, adding missing hydrogens/side chain atoms, and assigning the appropriate charge and protonation state for the acidic as well as basic amino acid residues at pH 7.0. The enzyme structure was subjected to an energy minimization step using the OPLS-2005 force-field^15,16^ with a root mean square deviation (RMSD) cut-off value of 0.30 Å to relieve the steric clashes among the residues due to the addition of hydrogen atoms^17^.

The preparation of the crystalized inhibitor, αk-13b, and the 75 candidate compounds were performed using LigPrep module of Schrodinger Suite which undertakes hydrogens atom addition, amending realistic bond lengths and angles, accurate chiralities, ionization states, tautomers, stereo chemistries, and ring conformations. Partial charges were assigned to the structures using the OPLS-2005 force-field^15^ and the subsequent structures were imperiled to energy minimization until their average RMSD reached to 0.001 Å. The ionization state was set at the neutral pH = 7 using Epik ionization tool^18^.

### Preparation of inhibitor-like ligand library

The 75 candidate compounds were retrieved from the experimental work proposed by Gordon et al*.*^13^ based on their experimental anti-viral activities against SARS-CoV-2. All candidate inhibitors were considered for further virtual screening-based 3D-pharmacophore features analysis. The library of the 75 compounds are presented in Table S1.

### Identification of 3D-pharmacophore hypotheses

For the structure-based pharmacophore modeling Schrodinger PHASE module^19^ was used with the default set of seven chemical features- hydrogen bond acceptor (A), hydrogen bond donor (D), hydrophobic contacts (H), negative ionizable (N), positive ionizable (P), and aromatic ring (R) to create the utmost illustrative features based on the crucial interactions with the key residues of the enzyme accommodated the inhibitor. The seven 3D-features were generated using Hypothesis Generation for Energy-Optimized Structure Based Pharmacophores considering the omitted volumes within 5 Å of the refined ligand for the enzyme^20^. The extracted pharmacophore hypothesis comprise the functional groups included in their bioactivity of targeted enzyme.

### Screening of M^pro^ inhibitors

All acquired seven 3D-pharmacophore features were exported and used for PHASE-based virtual screening of the 75 compounds library retrieved from recent experimental work by Gordon et al.^13^. Out of 75 candidates, 43 hit compounds were generated based on the highest PHASE screen score and matched ligand sites (Table S2). Both the quantity and quality of feature matching is taken into account in the Phase-Screen-Score.

### Docking-based virtual screening

Molecular-docking-based virtual screening was performed using Glide workflow of Maestro 11.6 to prioritize the lead compounds that strongly bind to M^pro^ enzyme^21^. Receptor grid was created as center coordinates (X = 9.81 Y = − 1.47 Z = 20.51) using two cubic boxes having a mutual centroid to systematize the calculations: a larger enclosing and a smaller binding box with dimensions of 24 × 24 × 24 Å and 18 × 18 × 18 Å, correspondingly. The grid box was centered on the centroid of the ligands in the complex, which was adequately large enough to search a superior region of the enzyme structure. All the chosen ligands were docked by using a three docking protocols of Glide^21^ which starts with “High throughput Virtual Screening” (HTVS) followed by “Standard Precision” (SP) and then by “Extra-Precision” mode (XP). Finally, the 43 input compounds were assessed using Docking-Based Virtual Screening and filtered to final three optimized lead compounds based on XP-GScores.

### ADMET properties assessment

Schrodinger QikProp 5.6 module was used to calculate absorption, distribution, metabolism, excretion and toxicity (ADMET) properties of the considered compounds to produce the ADMET associated descriptors. This protocol predicts noteworthy physicochemical and pharmacokinetic-based descriptors based on Lipinski’s rule of five^22,23^. ADMET properties of the top three compounds and crystalized control inhibitor were analyzed using QikProp 5.6 module and the best three compounds were considered for final analysis step through molecular dynamics (MD) simulations.

### MD simulations

MD simulation considered to be the most essential approach in understanding the fundamental structure and function of biological macromolecules. This method helps in finding the underlying dynamics and how it is connected to enzyme’s biomolecular function^24,25,26^. AMBER 18^27^ simulation package was used to execute 200 ns MD simulations on all the prepared complexes using (Graphics Processing Unit) GPU accelerated version of Partial Mesh Ewald Molecular Dynamics (PMEMD) simulations^28^. The ff99SB^29^ and the general AMBER force fields (GAFF)^30,31^ were employed to parametrize the enzyme and the considered ligands using LEaP implemented in Amber 18.

The ANTECHAMBER module was used to assign atomic partial charges for the ligands employed in General Amber Force-Field (GAFF). The system was solvated using the TIP3P^32^ explicit water in a cubic box with 8 Å box edge. The Na^+^ counter ions were added to randomly to neutralize the complex. The partial Mesh Ewald (PME)^33^ method was used to account the long-range electrostatic forces using cutoff of 12 Å, and the SHAKE algorithm^34^ was used to constrain all the hydrogen atoms bonds.

Energy minimizations were performed in two stages with 2500 steps of steepest decent minimization followed by 2500 of conjugated gradient to remove the bad contacts. The first stage was followed with a harmonic restraint of 500 kcalmol^−1^ A^−2^ on the solute molecule whereas, ions and water molecules were relaxed. On the second stage of minimization the restraints were removed and the whole system was relaxed. Each minimized complex was then gradually heated up from 0 to 300 K for 200 ps to keep the solute using a weak harmonic restraint of 10 kcalmol^−1^ A^−2^. The 50 ps density equilibration with weak restraints followed by the 500 ps constant pressure equilibration at 300 K were performed at constant pressure using Berendsen barostat^35^. Ultimately, the production phase of 200 ns MD simulation was performed on all the complexes at a constant temperature of 300 K and constant pressure of 1 atm^36^.

#### Post-dynamic trajectories analyses

The 200 ns MD trajectories were analyzed to calculate the RMSD of C^α^ atoms, root mean square fluctuation (RMSF) of each residue in the complex, radius of gyration (*R*_g_), solvent accessible surface area (SASA), and intramolecular/intermolecular hydrogen bond interactions using CPPTRAJ module^37^ implemented in AMBER 18. Molecular visualizations and plotting were conducted using Maestro 11.6 and OriginPro 2018 software^38^.

#### Principal component analysis (PCA)

PCA as an important tool for identifying the conformational changes of proteins was carried to describe the residual motions upon inhibitor binding of biomolecular complex^39^. PCA generates highly correlated and anti-correlated fluctuations derived from MD trajectories by applying dimensional reduction^40,41^. The collective motions were studied using the positional covariance matrix C constructed based on the atomic coordinates and their corresponding eigenvectors. The eigenvalues and eigenvectors are defined as the extent and the direction of motions, respectively^42,43^. By the following equation, the matric elements of the positional covariance matrix C were determined:

1 $$C_{i} = \left\langle {\left( {q_{i} - \left\langle {q_{i} } \right\rangle } \right)\left( {q_{j} - \left\langle {q_{j} } \right\rangle } \right)} \right\rangle \left( {i,j = 1,2, \ldots ,3N} \right)$$

where q_i_ and q_j_ are the cartesian coordinates for the i, jth of Cα atom, and N is the number of Cα atoms. To remove all translational and rotational movements, the average is calculated after superimposition with a reference structure using a least-square fit procedure to excerpt the important motion from MD trajectories^44,45,46^. To derive the eigenvalues and eigenvectors, the symmetric matrix C is transformed into a diagonal matrix Λ of eigenvalues by an orthogonal coordinate transformation matrix T:

2 $$\Lambda = T^{T} C_{ij} T$$

in which the eigenvectors correspond to the direction of motions relative to $\left\langle {q_{i} } \right\rangle$ and each eigenvector associate with an eigenvalue that represents the total mean-square fluctuation of the system along the corresponding eigenvector. CPPTRAJ module from the Amber 18 suite was used to perform the PC analysis and the porcupine plot of protein collective motions was created by NMWiz implemented in VMD^47^.

#### Binding free energy calculations

The relative binding free energies were computed using Molecular Mechanics/Generalized Born Surface Area (MM/GBSA) binding free energy method^48^. Water molecules and counter ions were stripped using the CPPTRAJ module. The binding free energies (Δ*G*_bind_) were calculated with the MM-GBSA method for each complex as below:

3 $$\Delta G_{bind} = G_{complex} - G_{protein} - G_{ligand}$$

4 $$\Delta G_{bind} = \Delta E_{gas} + \Delta G_{solvation} - T\Delta S$$

5 $$\Delta E_{gas} = E_{int} + E_{vdw} + E_{elec}$$

6 $$E_{int} = E_{bond} + E_{angle} + E_{torsion}$$

7 $$G_{solvation, GB} = G_{GB} + G_{nonopolar, solvation}$$

8 $$\Delta G_{nonpolar} = \gamma SASA + \beta$$

The gas phase energy (Δ*E*_gas_) is the sum of the internal (*E*_int_), van der Waals (*E*_vdW_) and Coulombic (*E*_elec_) energies, (Eq. 6). The solvation free energy is the pattern of polar (G_GB_) and nonpolar (Gnonpolar, solvation) contributions (Eq. 7). The polar solvation G_GB_ contribution is estimated using the Generalized Born (GB) solvation model with the dielectric constant 1 for solute and 80.0 for the solvent. Conversely, the nonpolar free energy contribution was assessed using Eq. 8, where the surface tension proportionality constant, γ, and the free energy of nonpolar solvation of a point solute, β, were set to 0.00542 kcal mol^−1^ Å^−2^ and 0 kcal mol^−1^, respectively^49^. The SASA is calculated by the linear combination of pairwise overlap (LCPO) model^50^.

## Result and discussion

### Selection of compounds

An specific drug repurposing library of 75 anti and pro-viral agents including FDA approved drugs, clinical trials compounds and preclinical compounds with enzyme inhibitory activity between 10 and 100 nM range^13^ against SARS-CoV-2 was considered as the input library for this in silico study. The library including the compounds name as well as their corresponding smile structures are presented in Table S1.

### Structure-based pharmacophore modeling

**Figure 2:**
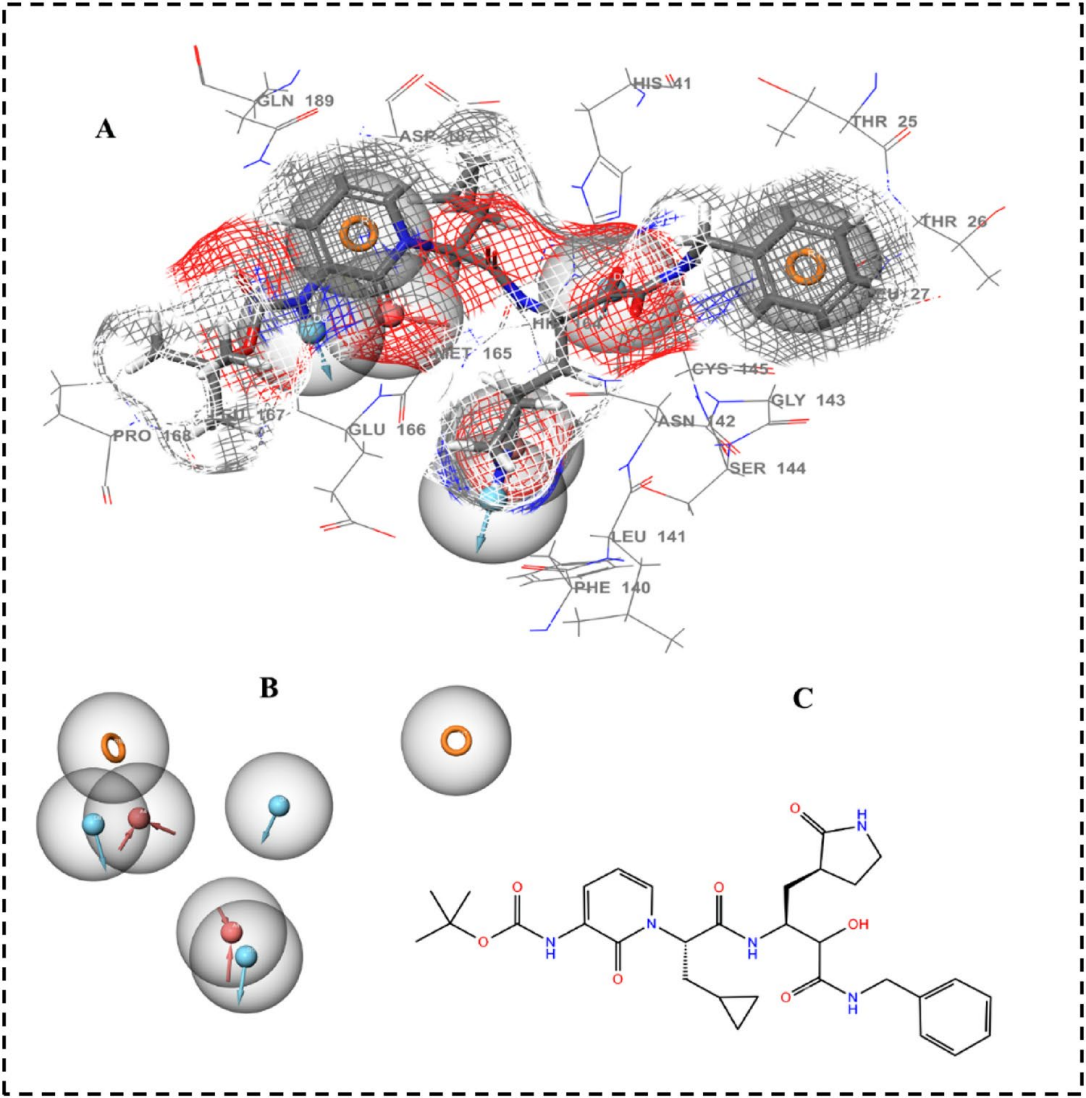
(**A**) 3D structure-based pharmacophore features of αk-13b inhibitor in the complex interacting with M^pro^ binding site. (**B**) The seven generated pharmacophore features in Red arrow: Hydrogen bond acceptor, blue arrows: Hydrogen bond donor, orange: aromatic ring. (**C**) 2D chemical structure of the inhibitor.

A comprehensive and accurate information of ligand interacting features can be obtained from structure-based pharmacophores based on three-dimensional structure of a target protein^51^. The most common descriptors in pharmacophore modeling are H-bond donors, H-bond acceptors, positive and negative ionizable groups, lipophilic regions and aromatic rings. The most effective 3D structure-based e-pharmacophores were produced using the receptor–ligand pharmacophore generation protocol implemented in PHASE, which was executed for a co-crystal αk-13b inhibitor inside the active pocket in order to determine possibly critical amino acids that are involved in ligand binding (Fig. 2A). The generated e-pharmacophore for the considered enzyme showed seven main 3D-features including, H-bond acceptor, H-bond donor and π–π stacking of aromatic ring. In each pharmacophore feature, the red arrows represent hydrogen bond acceptor, blue arrow represents hydrogen bond donor and orange spheres represent π–π stacking of aromatic ring, Fig. 2B. Numerous excluded volumes were also produced in the models to demonstrate the space balancing. The seven 3D pharmacophore features and 2D-chemical structure of αk-13b inhibitor are presented in Fig. 2B,C showing three donor hydrogen bonds, three acceptor hydrogen bonds and one aromatic ring sphere.

### Virtual screening of the candidate compounds

The obtained structure-based pharmacophore hypotheses of αk-13b inhibitor in complex with M^pro^ were used to screen the 75 candidate anti-viral compounds retrieved from recent experimental work by Gordon et al.^13^ (Table S1). These compounds were screened based on PHASE screen score, matched ligand sites indices. A total of 43 compounds subsequently passed this filter based on the created pharmacophore hypothesis. Molecules which have satisfied all the features of the pharmacophore hypothesis were considered as potential hits. The output of virtual screening analysis of 43 compounds consist of their PHASE screen score and matched ligand sites are presented in Table S2.

### Docking-based virtual screening analysis

**Figure 3:**
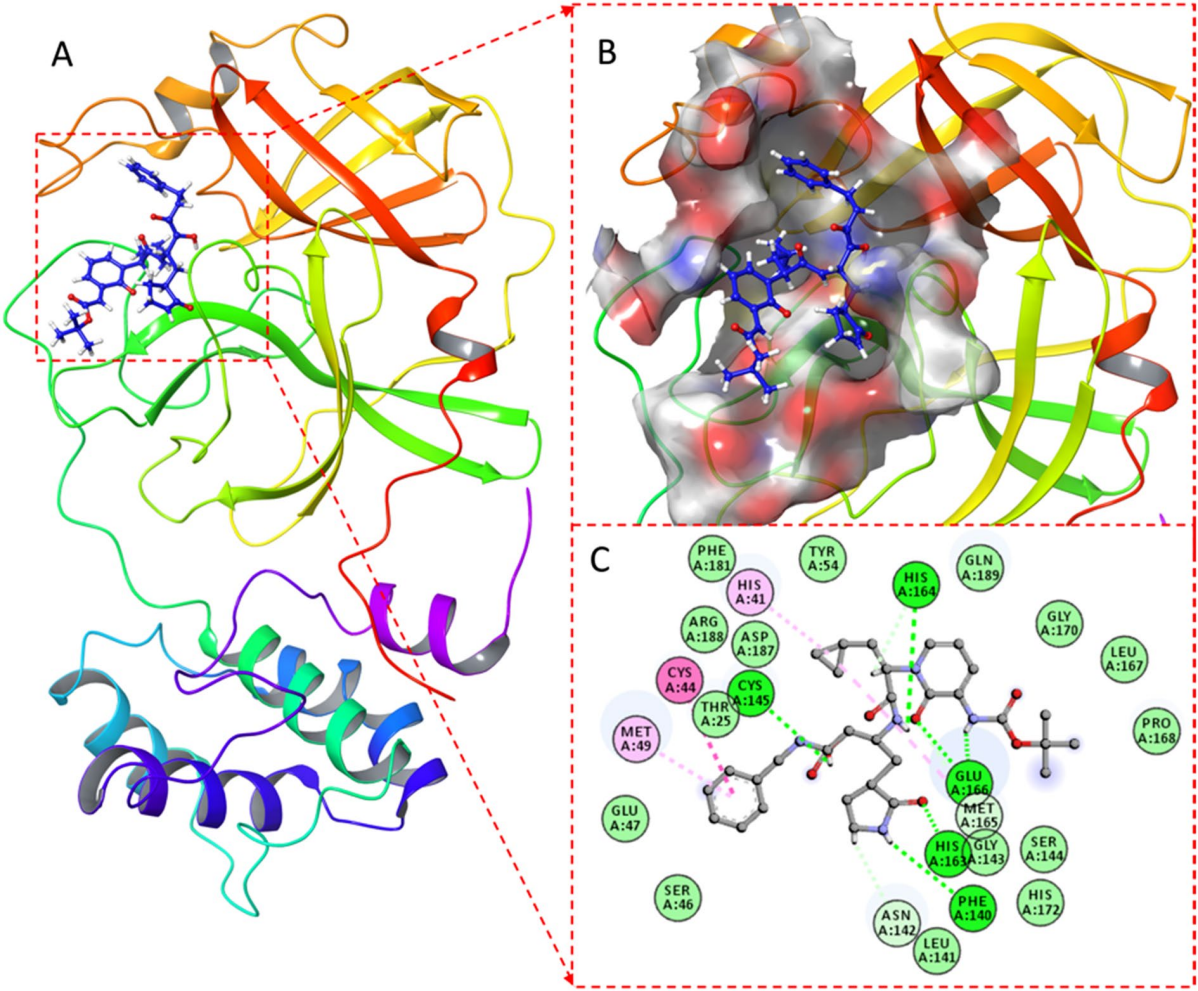
(**A**) αk-13b inhibitor in complex with M^pro^ enzyme, (**B)** Surface view colored by charge showing the catalytic pocket of the Mpro enzyme; (**C**) 2D representation of the interaction map of docked inhibitor in complex with M^pro^.

**Figure 4:**
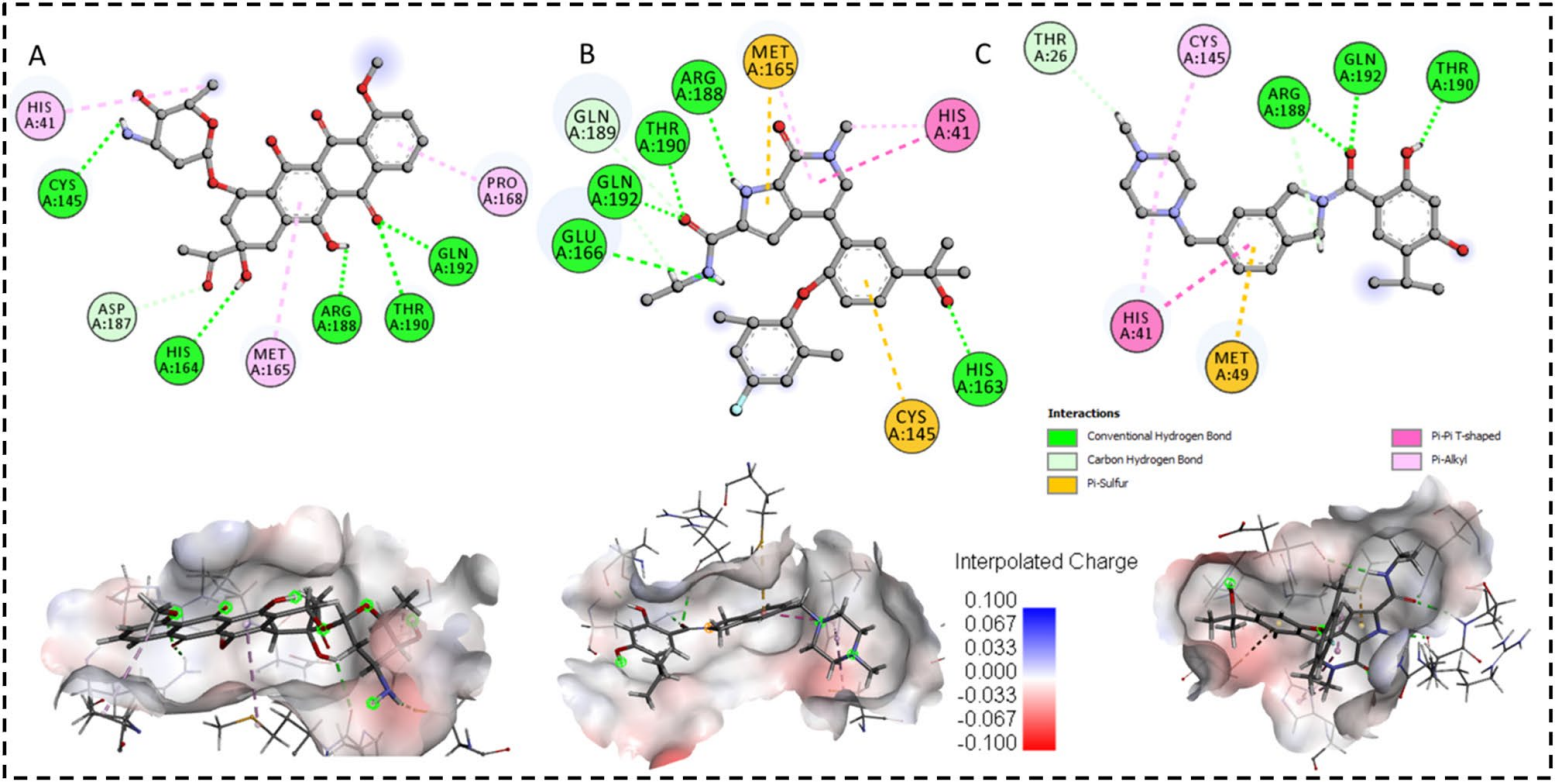
Docked poses of M^pro^ enzyme with the proposed inhibitors. 2D plots and binding interactions of M^pro^ enzyme with compounds (**A)** Daunorubicin, **(B)** ABBV-744 and (**C)** Onalespib. Lower panels are representing the surface view of conserved substrate-binding pocket of M^pro^ in complexed with Daunorubicin, ABBV-744 and Onalespib, respectively (left to right).

The 43 screened compounds obtained from virtual screening were considered for docking analysis using Glide workflow^52^ of Schrödinger package. Three step wise filtering protocol were used for docking using HTVS where a total of 23 compounds (Table S3) were obtained followed by Glide SP where a total of 12 hits were generated (Table S4). Finally, the best lead compounds were obtained using Glide XP lead optimization protocol, while among 10 generated docking poses per ligand, only one pose was retained (Table S5). The Glide GScore and the interacting binding residues of the five lead compounds presented in Table 1. The αk-13b inhibitor as well as the best optimized lead generated from XP docking were selected to map their potential interactions within the active pocket of SARS-CoV-2 M^pro^ enzyme using molecular docking approach. This approach aids in understanding the optimized orientation of a ligand and its target protein by minimizing inclusive energies of the corresponding complexes. The estimated docking binding energy values of all three compounds Daunorubicin, Onalespib and ABBV-744 with their experimentally viral inhibition activity (pIC50) 6.67, 6.81, 2.46 against SARS-CoV-2^13^ as well as αk-13b inhibitor are shown in Figs. 3 and 4 and Table 1.

**Table 1:** The best three compounds generated using XP docking with their corresponding docking scores and the contributing binding residues are presented. Catalytic dyad residues are shown in bold.

Compounds	Glide GScore kcal/mol	Contributing binding residues
Daunorubicin	− 9.33	ASP187, ARG188, GLN189, THR190, ALA191, GLN192, MET49, TYR54, **HIS41**, VAL142, **CYS145**, GLY143, HIS163, HIS164, MET165, GLU166, PRO168, CYS44, VAL168
Onalespib	− 8.21	VAL186, **HIS41**, **CYS145**, SER144, GLY143, ASN142, LEU27, THR26, THR25, MET49, LEU50, ASP187, ARG188, GLN189, THR190, ALA191, GLN192, PRO168, LEU167, GLU166, MET165, HIS164
ABBV-744	− 7.79	**HIS41**, MET49, **CYS145**, SER144, GLY143, ASN142, LEU141, PHE140, HIS163, HIS164, HIE172, MET165, GLU166, LEU167, PRO168, ASP187, ARG188, GLN189, THR190, GLN192,
αk-13b inhibitor	− 6.75	HIE172, PHE140, LEU141, ASN142, GLY143, SER144, **CYS145**, GLN189, ARG188, ASP187, **HIS41**, CYS44, MET49, TYR54, PRO168, LEU167, GLU166, MET165, HIS164, HIS163, THR25, PHE181, SER46, GLU47, GLY170

As it is shown in Fig. 3, αk-13b inhibitor interacted to HIS164, GLU166, HIS163, PHE140, ASN 142 and CYS145 through six hydrogen bond interactions. HIS41, CYS44 and MET49 formed three π–π stacking interaction with αk-13b inhibitor.

The Daunorubicin-M^pro^, Onalespib-M^pro^ and ABBV-744-M^pro^ docked complexes presented considerable binding affinities with the energy values of − 9.33, − 8.21 and − 7.79 kcal mol^−1^, respectively (Table 1). These three lead compounds contributed into the binding site of M^pro^ enzyme through the hydrogen bonding, π–π stacking and π-Sulphur interactions (Fig. 4).

The daunorubicin created one hydrogen bond and one π-alkyl interaction with the catalytic dyad CYS145 and HIS41, respectively. The other five hydrogen bonds were formed by the hydroxyl (-OH) groups of daunorubicin with HIS164, Arg188, Thr190, ASP187 and Gln192 as presented in Fig. 4A. ABBV-744 formed the interaction network of six hydrogen bonds with Arg188, Thr190, GLU166, HIS163, GLN189 and Gln192 with the hydroxyl group (-OH) of the compound, Fig. 4B. The catalytic dyad CYS145, HYS41 and MET165 formed π–π stacking interaction and π-Sulphur interactions with ABBV-744. Onalespib formed an interaction network of total four hydrogen bonds with THR26, ARG188, THR 190 and GLN192. The catalytic dyad and MET165 interacted to Onalespib through π–π stacking and π-sulphur interactions (Fig. 4C).

Thus, it could be contemplated that these three compounds bound favorably to the binding site of M^pro^ through hydrogen bond, π–π stacking and π-alkyl interactions mainly generated by CYS145, HIS41, MET165, HIS163, GLU166, GLN 189, Arg188, Thr190 and Gln192 as key contributing active residues into the docked complexes.

### ADMET analysis

Pharmacokinetic and toxicity features were predicted using QikProp module of Schrodinger for Daunorubicin, Onalespib, ABBV-744 and αk-13b inhibitor. Outcomes of pharmacokinetic and toxicity study are illustrated in Table 2. The selected properties of the compounds are representatives of influence metabolism, cell permeation, bioavailability and toxicity.

**Table 2:** In-silico ADMET screening of the selected compounds.

Compounds	^a^CNS	^b^QPlogKhsa	^c^SASA	^d^QPlogPo/w	^e^QPlogS	^f^QPlogBB	^g^%Human oral absorption	^h^Rule of five
Daunorubicin	− 2	− 0.32	716.74	0.49	− 2.30	− 1.88	9.34	3
Onalespib	1	0.58	763.23	3.03	− 3.92	− 0.3	74.42	0
ABBV-744	− 2	0.95	798.91	4.83	− 7.15	− 1.42	100.0	0
αk-13b inhibitor	− 2	− 0.07	960.42	3.11	− 5.15	− 2.91	33.40	3

^a^Predicted central nervous system activity from − 2 (inactive) to + 2 (active). ^b^Prediction of binding to human serum albumin (acceptable range: − 1.5–1.5). ^c^Total Solvent Accessible Surface Area: SASA (acceptable range: 300–1000). ^d^Predicted octanol/water partition coefficient (acceptable range: − 2–6.5). ^e^Predicted aqueous solubility, S in mol/dm^−3^ (acceptable range: − 6.5–0.5). ^f^Predicted brain/blood partition coefficient (acceptable range: − 3.0–1.2). ^g^Predicted percentage human oral absorption (< 25% is poor and > 80% is high). ^h^Number of violations of Lipinski’s rule of five, Compounds that satisfy these rules are considered druglike (maximum 4).

The predicted central nervous system activity (CNS) of Daunorubicin, ABBV-744 and αk-13b inhibitor depicted as inactive whereas, Onalespib was presented as an active compound. The predicted human binding serum albumin (QPlogKhsa) of all compounds showed in the acceptable range. The estimated total solvent accessible surface area (SASA) of all three compounds and αk-13b inhibitor met the acceptable range: 300–1000. Predicted octanol/water partition coefficient (QPlogPo/w) showed in the acceptable range from − 2 to 6.5 for all the ligands. The predicted aqueous solubility (QPlogS) for Daunorubicin, Onalespib and αk-13b inhibitor were in the acceptable range of − 6.5–0.5 whereas ABBV-744 showed slightly low value. The predicted brain/blood partition coefficient (QPlogBB) for all these compounds showed in the acceptable ranges. The percentage human oral absorption for all the compounds met in the recommended range. Number of violations of Lipinski’s rule of five for all the compounds satisfied this rule for all the studied ligands.

### Post-dynamics MD trajectories analysis

The structural variations within the enzymes structure is correlated with their biological activities. Any alterations or interference on enzymes structural integrity might have a substantial impact on its activity^41^. The binding of inhibitors influence the mode of action of enzymes that are comprised in disease pathways, thus there is a requirement to estimate the structural dynamics and conformational changes associated with the inhibitory activity of these inhibitors^53^.

**Scheme 1:**
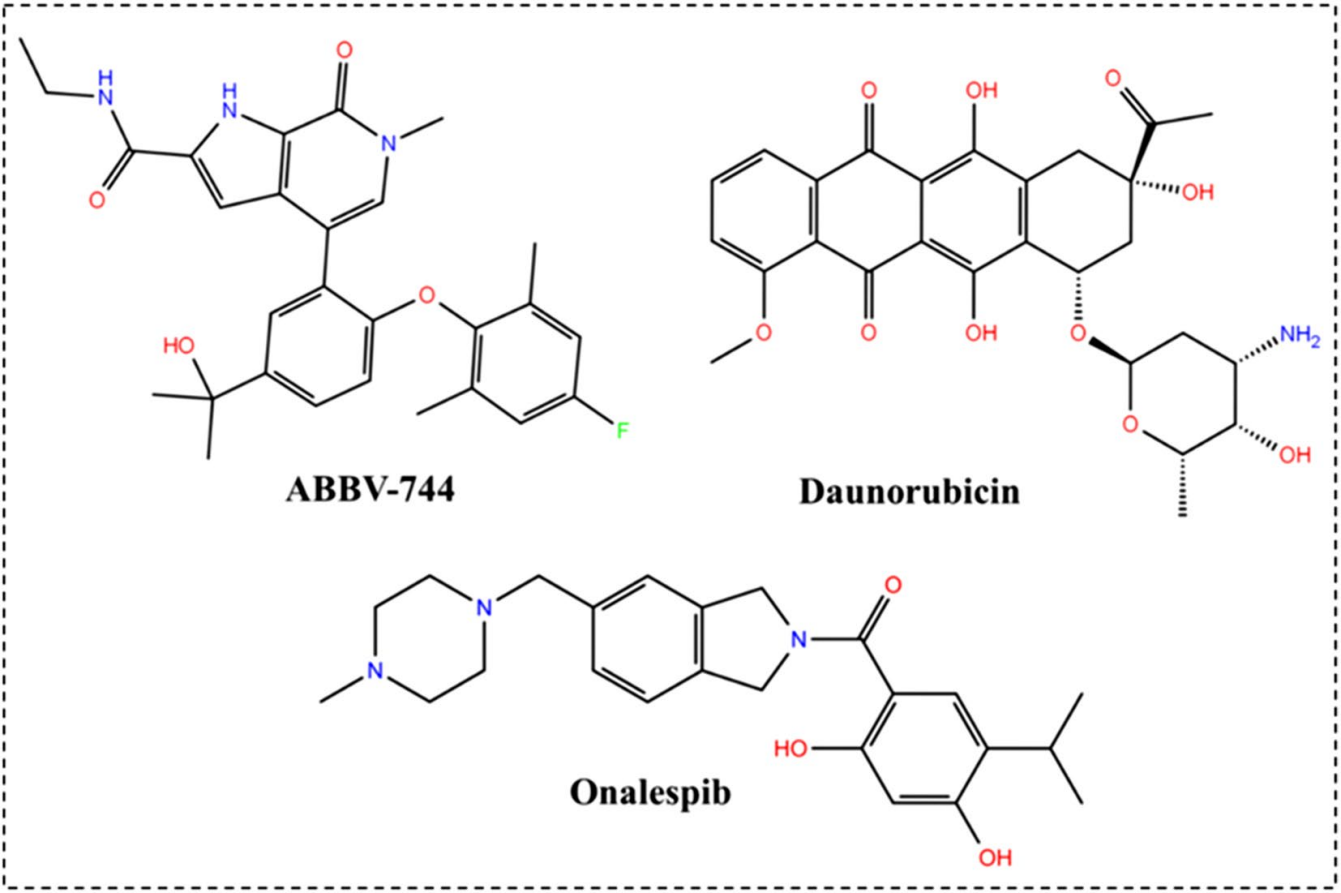
2D chemical structure of top three hit compounds.

In this section, 200 ns MD trajectories regarding the four complexes, namely, Daunorubicin-M^pro^, Onalespib-M^pro^, ABBV-744-Mpro and αk-13b inhibitor-M^pro^ as control model were analyzed. Different metrics and analysis were applied to investigate the stability and flexibility of the complexes as well as the contribution of the studied compounds upon binding in terms of binding free energies. The 2D chemical structure of all the ligands considered for MD simulations are presented in Fig. 1 and Scheme 1.

The computation of a time variable with reference to an RMSD of C_α_ atoms from generated trajectories was accomplished to investigate the consistency and efficiency of M^pro^ in complex with αk-13b inhibitor and along with the three lead compounds, Fig. 1 and Scheme 1.

**Figure 5:**
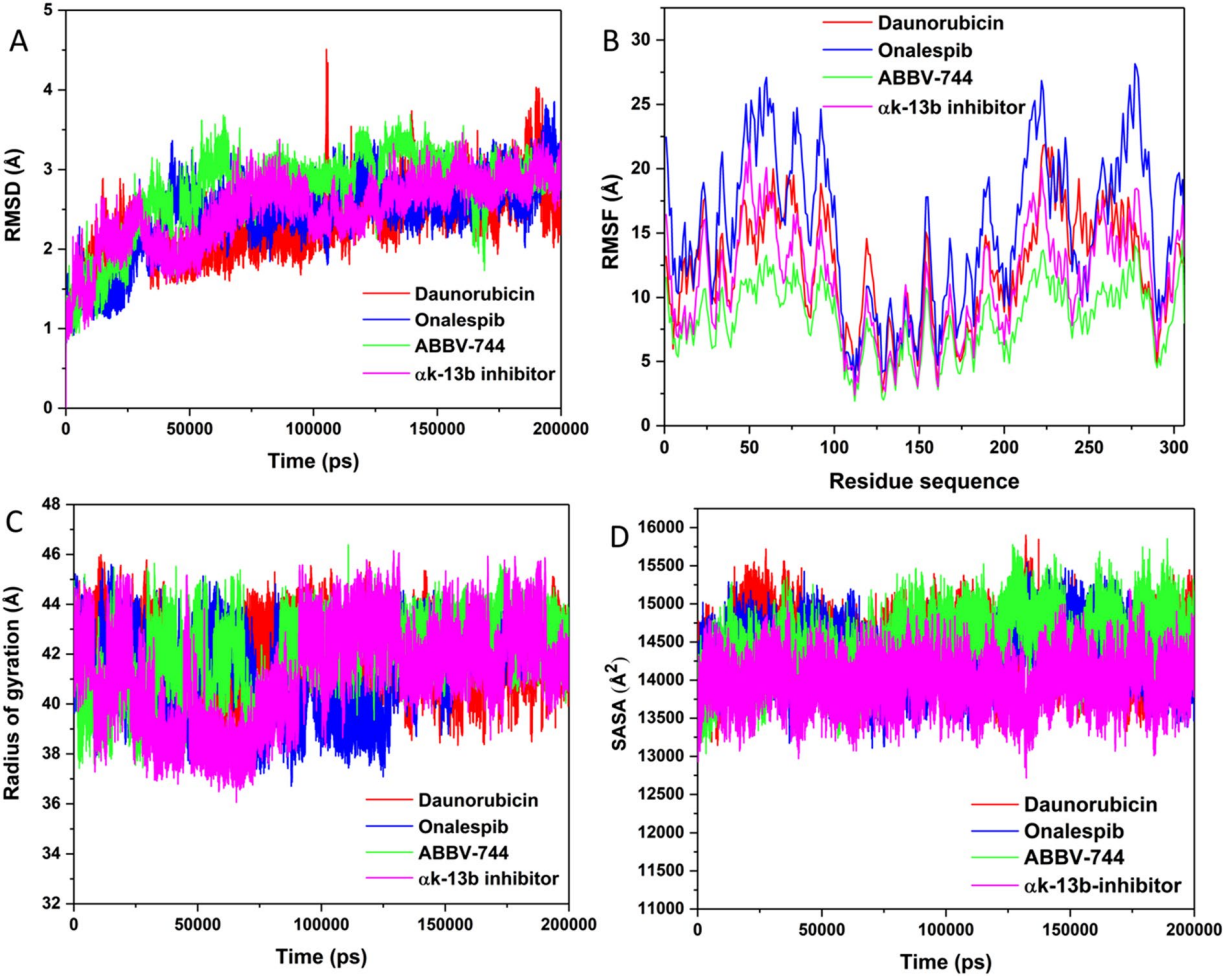
Structural dynamics of M^pro^ enzyme-ligand complexes (Daunorubicin in red, Onalespib in blue, ABBV-744 in green and αk-13b inhibitor in magenta) during 200 ns of MD simulations. (**A**) C^α^ backbone RMSD in Å of all the selected compounds bound to M^pro^ enzyme; (**B**) Values of RMSF in Å plotted against residue number for all the selected compounds bound to M^pro^ enzyme; (**C**) *R*_g_ values after compound binding; and (**D**) SASA values of C^α^ backbone atoms.

The perturbations in the RMSD values as denoted in plot (Fig. 5A) throughout the simulation time disclosed the possible conformational deviances in the enzyme structure upon ligand binding. As Fig. 5A revealed, all the complexes were stabilized and attained convergence after almost 50 ns of simulation run. ABBV-744-M^pro^ unveiled the lowest average RMSD of 2.45 Å, while Onalespib-M^pro^ and Daunorubicin-M^pro^ revealed average RMSD of 2.73 Å and 2.76 Å respectively. The αk-13b inhibitor-M^pro^ unveils a perturbation of 2.85 Å as indicated in the plot. This evaluation proposed that any further analyses performed on the produced trajectories of all complexes were reliable. The RMSD plots showed that ABBV-744-M^pro^, Onalespib-M^pro^, Daunorubicin-M^pro^ and αk-13b inhibitor-M^pro^ complexes exhibit reasonable convergence indicating stability of the systems during the MD trajectories. The variation of ligand RMSD for ABBV-744-M^pro^ (0.04 Å), Onalespib-M^pro^ (0.17 Å) and Daunorubicin-M^pro^ (0.05 Å) complexes showed the considerable stability of the ligand position inside the binding pocket, Figure S1.

To provide detailed insight into the structural fluctuation and flexibility of different regions of the amino acid residues of M^pro^ enzyme upon binding of the selected compounds, RMSF values for Cα atoms were calculated from trajectories generated over 200 ns of MD trajectories. Hence, ligand binding to the enzyme could be investigated in relation to the modification in flexibility in terms of RMSF values^54^. To discover the stringency and elasticity in M^pro^ residues upon binding of chosen compounds, RMSF values for C^α^ atoms were estimated from trajectories produced from 200 ns of MD simulations run. As shown in Fig. 5B, ABBV-744-M^pro^ complex showed the least fluctuations in the amino acid residues with 7.56 Å. An average RMSF of 13.76 Å and 15.09 Å was spotted in complex Daunorubicin-M^pro^ and Onalespib-M^pro^, correspondingly. The complex, αk-13b inhibitor-M^pro^ disclosed an average of 14.11 Å that is remarkably greater than ABBV-744-M^pro^ complex, signifying enhanced binding in comparison to the αk-13b inhibitor-M^pro^ complex. This noteworthy decline might be coupled with structural inactivation that evidently confirmed as a result of significant binding of ABBV-744 compound in the active pocket of M^pro^ enzyme. The reduced fluctuation of amino acid residues might have favored M^pro^ enzyme inhibition through compound ABBV-744.

The radius of gyration (Rg) parameter was assessed as the structural compactness index and its folding and unfolding behavior through the overall conformational variations in enzyme structure upon inhibitor binding. The mediocre values of *R*_g_ for ABBV-744-M^pro^, Onalespib-M^pro^ and Daunorubicin-M^pro^ complexes were noted to be 41.55 Å, 41.87 Å and 42.08 Å, respectively. Figure 5C plots disclosed extremely minor changes in the compactness of the three compounds. The compound ABBV-744 exhibited a lowest *R*_g_ in comparison with other two complexes, and with the control αk-13b inhibitor-M^pro^ complex (42.36 Å). This observation proposed increased compactness and enhanced binding with the ABBV-744 M^pro^ and Onalespib-M^pro^ complexes. All these patterns of conformational analysis are suggesting an improved stability, flexibility and compactness of compound ABBV-744 in complex the M^pro^ enzyme.

Solvent Access Surface Area (SASA) analysis was performed to define the activity of hydrophobic and hydrophilic amino acid residues and forces exposed to the solvent over 200 ns MD trajectories. The constant and accurate scheming of SASA is highly useful in the energetic evaluation of biological macromolecules^55^. The interfaces among the hydrophobic native contacts inside enzyme structure is a noteworthy intermolecular interaction that effect enzyme inhibition. Hydrophobic interaction constructed between the non-polar residues corroborate the stability of the enzyme structure in solution by sheltering the non-polar residues inside the hydrophobic core distant from an aqueous solution^56^. As shown in Fig. 5D, standard SASA values for all selected compounds have been measured during 200 ns MD trajectories. Average value of SASA for the compound ABBV-744-M^pro^ complex is 14,230 Å^2^ which was showed to the solvent system. Overall SASA values of 14,303 Å^2^ and 14,426 Å^2^ were prominent by Onalespib-M^pro^ and Daunorubicin-M^pro^ complexes, individually. The differences in SASA values for all the complexes during the simulation period corresponds with the folding and unfolding of enzyme structure. The overall SASA values in the control complex was 14,001 Å^2^, slightly less than ABBV-744-M^pro^ complex. The SASA assessment perceived in compound ABBV-744 bound complex additionally validated that ABBV-744 compound has better exposure to solvent and consequently favored the improved inhibitory activity of compound ABBV-744 over other complexes.

#### Hydrogen bond analysis

**Figure 6:**
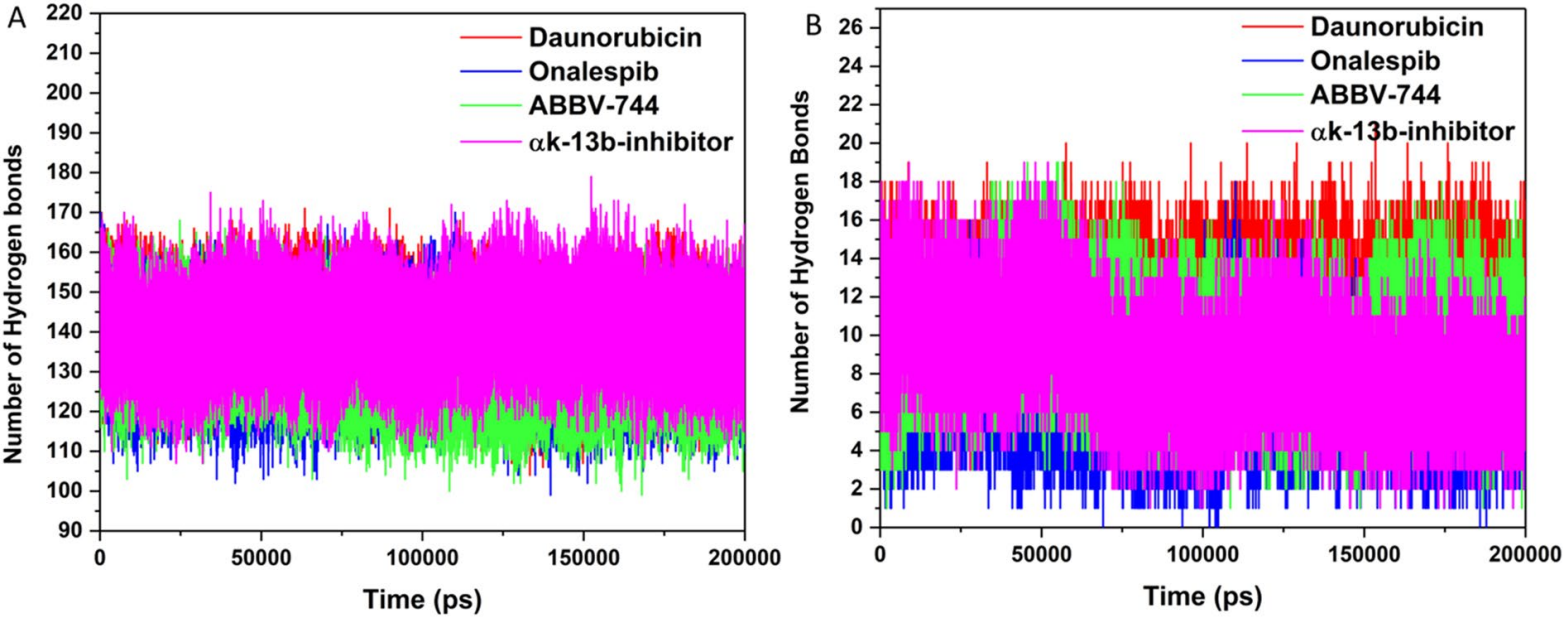
Hydrogen bond analysis. (**A**) Intramolecular and (**B**) Intermolecular hydrogen bonds in M^pro^ enzyme with the selected compounds (Daunorubicin in red, Onalespib in blue, ABBV-744 in green and αk-13b inhibitor in magenta) calculated after 200 ns MD simulation.

For overall conformation and stability of enzyme structure, we have measured the intramolecular and intermolecular hydrogen bond analysis (Fig. 6). This analysis gives extreme understanding into binding mechanism of enzyme-ligand with detailed consideration^57^. An average number of intramolecular hydrogen bonds in ABBV-744-M^pro^ complex was noted to be 136 as displayed in Fig. 6A. In compound Onalespib and Daunorubicin, the intramolecular hydrogen bonds were observed to be 139 and 140, respectively.

The number of intermolecular hydrogen bonds produced in the catalytic site of M^pro^ enzyme notable to be 9–10 in ABBV-744 bound M^pro^ complex. However, number of these bonds are more in Daunorubicin-M^pro^ complex with 11–12 hydrogen bonds and less in Onalespib and αk-13b-inhibitor bound M^pro^ with 7–8 hydrogen bonds as presented in Fig. 6B.

#### Principal component analysis

**Figure 7:**
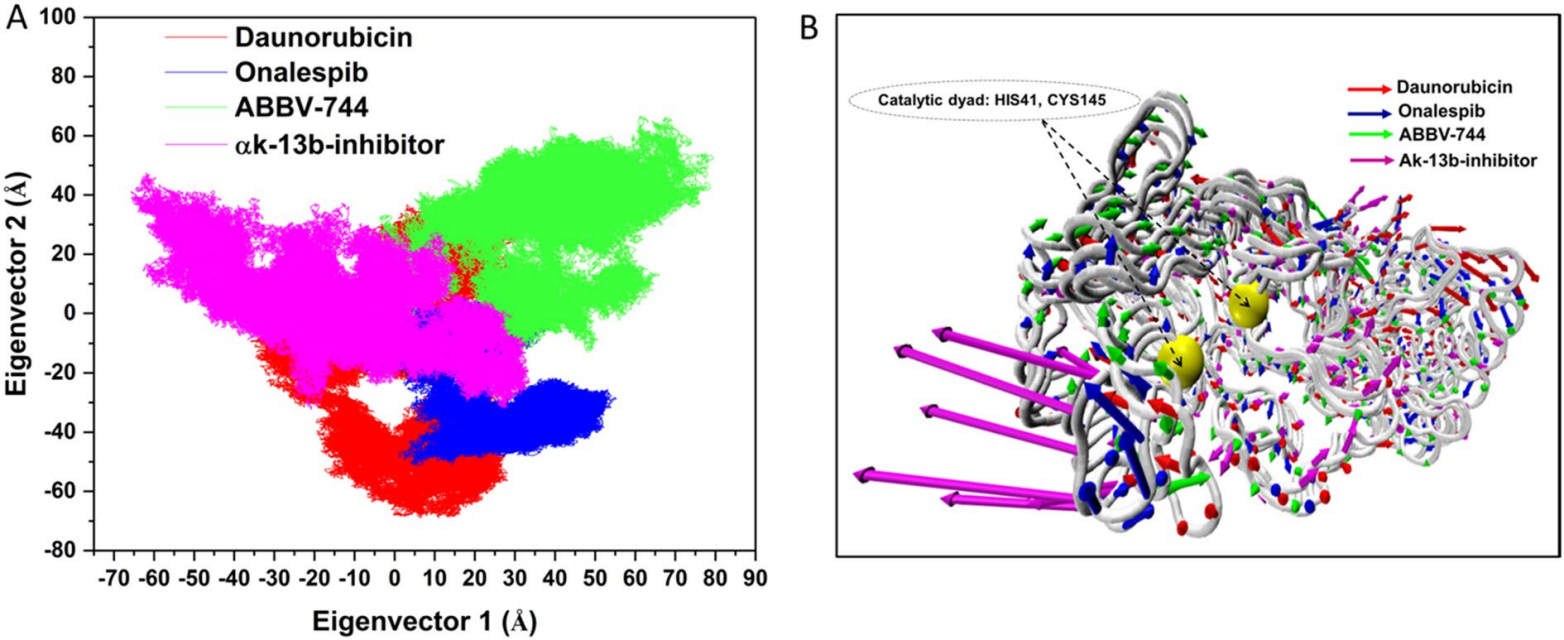
(**A**) PCA plot constructed by eigenvector 1 vs eigenvector 2 for Daunorubicin (red), Onalespib (blue), ABBV-744 (green) and αk-13b inhibitor (magenta) complexes. (**B**) PC1 collective motions for the obtained predominant eigenvectors using principal component analysis over the 200 ns MD trajectories for Daunorubicin-M^pro^, Onalespib-M^pro^, ABBV-744-M^pro^ and αk-13b inhibitor-M^pro^.

To qualitatively probe the impact of inhibitor’s binding on the predominant conformational motion of each residue^58,59^ , the concerted motions in Daunorubicin-M^pro^, ABBV-744-M^pro^ and Onalespib-M^pro^ and αk-13b inhibitor-M^pro^ complexes were studied using PC analysis based on the eigenvector. The scatter plots in Figure, essentially give a two-dimensional representation of the conformational changes occupied by the system. The gradual migration of the points in the PC1−PC2 scatter plots obtained using construction of eigenvectors, Fig. 7A. PC1 collective motions extracted for the predominant eigenvectors of the using principal component in the studied complexes, Fig. 7B.

The scatter plots of the complexes in Figure, indicate the undergoing overall motions of the protein upon binding in terms of correlated and anti-correlated movements, Fig. 7A,B.

It is evident in Fig. 7A, the Onalespib, Daunorubicin and αk-13b-inhibitor with the trace covariance matric of 37.45 Å^2^, 37.67 Å^2^ and 37.69 Å^2^, imposed highly fluctuated anti-correlated effects as the negative values of 2D-scatter points into the protein, Fig. 7B. Interestingly, in the case of ABBV-744 with the trace covariance matric of 37.64 Å^2^, the prominent correlated motions were observed with the least fluctuations of the system upon ligand binding, Fig. 7B. Thus, from the above observations Fig. 7B, it was concluded that ABBV-744 induced least fluctuations into the binding site upon binding than the variants complexes.

#### Mechanistic insights into binding affinity

To understand the impact of inhibitors upon complexation in terms of their binding affinities, MM-GBSA binding free energy method were utilized to calculate the binding free energies and their energy components of the complexes, Table 3.

**Table 3:** Binding free energies and its components for the three hit compounds:M^pro^ and αk-13b inhibitor:M^pro^ using MM-GBSA method. The energy components are in kcal mol^−1^.

Complex	Δ*E*_vdw_	Δ*E*_elec_	Δ*G*_gas_	Δ*G*_polar_	Δ*G*_nopolar_	Δ*G*_solvation_	Δ*G*_bind_
Daunorubicin-M^pro^	− 49.65	− 4.39	− 54.05	22.48	− 5.09	17.39	− 36.65
Onalespib-M^pro^	− 43.55	− 18.55	− 62.10	29.42	− 4.45	24.97	− 37.13
ABBV-744-M^pro^	− 54.45	− 35.20	− 89.65	50.03	− 5.81	44.22	− 45.43
αk-13b inhibitor-M^pro^	− 36.35	− 8.14	− 44.49	28.21	− 4.64	23.57	− 20.92

As it is evident in Table 3, the total binding free energies (Δ*G*_bind_) of Daunorubicin-M^pro^, Onalespib-M^pro^, ABBV-744-M^pro^ and αk-13b inhibitor-M^pro^ were − 36.65 kcal/mol, − 37.13 kcal mol^−1^, − 45.43 kcal mol^−1^ and − 20.92 kcal mol^−1^, respectively. Accordingly, among all the studied complexes, ABBV-744-M^pro^ and Onalespib-M^pro^ depicted the most favorable of Δ*G*_bind_ with lowest values of − 45.43 kcal mol^−1^ and − 37.13 kcal mol^−1^. At this point, it is interesting to address the key contributions that each binding component can impose to the total binding free energies.

It is evident that amongst the studied complexes, the Δ*G*_gas_ as the favorable contributing index into the total Δ*G*_bind_ has the lowest values for ABBV-744-M^pro^ (− 98.65 kcal mol^−1^) and Onalespib-M^pro^ (− 62.10 kcal mol^−1^) complexes. This observation implies the most favorable contribution values of ΔE_vdw_ and ΔE_elec_ for ABBV-744-M^pro^ (− 54.45 kcal mol^−1^ and − 35.20 kcal mol^−1^) and Onalespib-M^pro^ (− 43.55 kcal mol^−1^ and − 18.55 kcal mol^−1^) into the total binding free energies.

Interestingly, this observation revealed another key contributing component of ΔG_nonpolar_ for the complexes of ABBV-744-M^pro^ (− 5.81 kcal mol^−1^) and Onalespib-M^pro^ (− 4.45 kcal mol^−1^) which leads to the lower ΔG_bind_ values for both complexes. According to the obtained energy components results, it could be inferred that the ΔE_vdw_, ΔE_elec_ and ΔG_nonpolar_ have the dominant contribution into the binding affinities for the selected complexes.

#### Per-residue decomposition energy analysis

**Figure 8:**
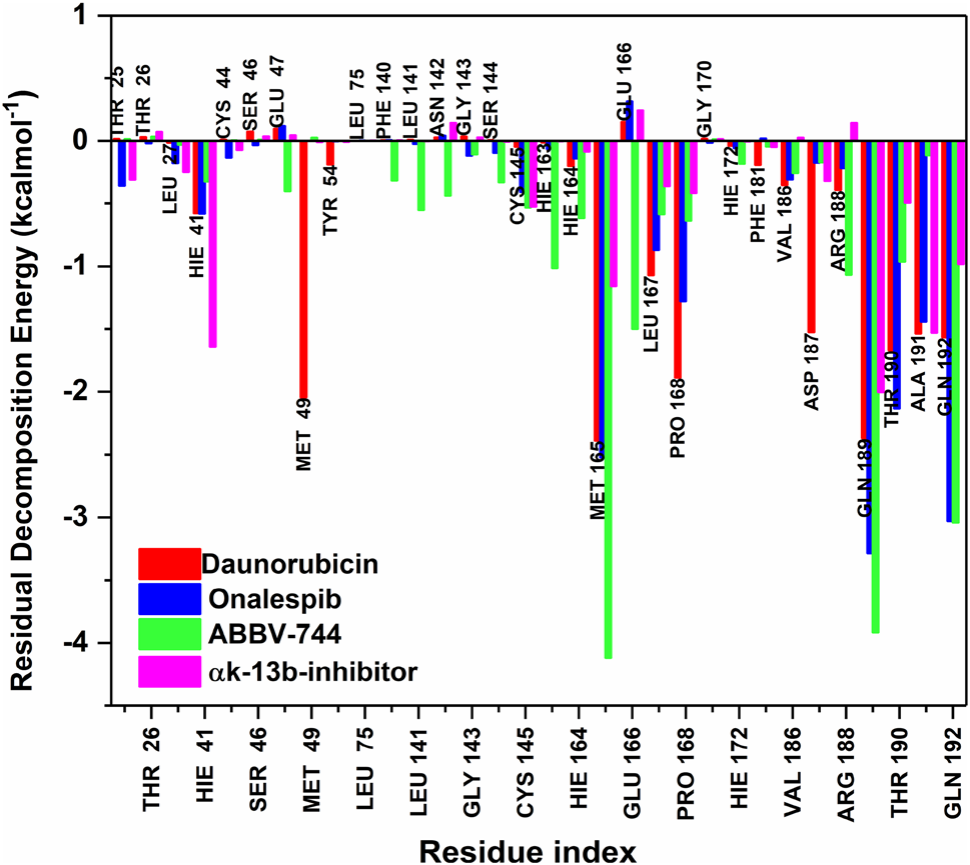
Per residual decomposition energy of selected compounds (Daunorubicin in red, Onalespib in blue, ABBV-744 in green and αk-13b inhibitor in magenta) bound to M^pro^ enzyme calculated with MMGB/SA approach.

The binding-free energy decomposition offers an immense understanding in account of enzyme-ligand complexes produced from the trajectories by MD simulations. To achieve this, we fragmented the total binding energies of complexes into each-residual involvement by per amino acid residue existing in the catalytic site of M^pro^ enzyme to provide comprehensive identification of key contributing residues upon ligand binding as depicted in Fig. 8. The interactions between catalytic site electro-negative and electro-positive residues develops ligand binding and its stabilization at the target enzyme. This creates an improved intermolecular binding that surges the binding affinity of the ligand in the active site. Active site residue MET165 in ABBV-744 contributed with the lowest Δ*G*_bind_ with − 4.22 kcal mol^−1^ however this residue has contributed with notably less Δ*G*_bind_ of − 2.50 kcal mol^−1^ and − 2.45 kcal mol^−1^ in Onalespib and Daunorubicin bound complexes. The Δ*G*_bind_ of another participating residues GLN189 was also observed to be lowest in ABBV-744 bound M^pro^ complex with − 3.94 kcal mol^−1^ however, it is slightly less in Onalespib-M^pro^ complex with value of − 3.45 kcal mol^−1^ and − 2.48 kcal mol^−1^ in Daunorubicin-M^pro^ complex. Gln192 significantly contributed in the binding of ABBV-744 compound with Δ*G*_bind_ value of − 3.00 kcal mol^−1^ and observed a very minor difference with − 2.99 kcal mol^−1^ Δ*G*_bind_ in Onalespib bound M^pro^ but showed a lesser Δ*G*_bind_ of − 1.50 kcal mol^−1^ in Daunorubicin compound.

GLU47, PHE140, LEU141, ASN142, GLY143, SER144 and GLU166 are the other active site amino acid residues which favored with the negative binding of ABBV-744 compound with Δ*G*_bind_ values of − 0.48 kcal mol^−1^, − 0.39 kcal mol^−1^, − 0.52 kcal mol^−1^, − 0.50 kcal mol^−1^, − 0.14 kcal mol^−1^, − 0.41 kcal mol^−1^ and − 1.50 kcal mol^−1^ respectively, however, these residues contributed with positive energies in Onalespib and Daunorubicin bound M^pro^ complexes. The catalytic dyad residues, His41 and Cys145 showed the lowest Δ*G*_bind_ value of − 1.64 kcal mol^−1^ and − 0.61 kcal mol^−1^ in control inhibitor αk-13b inhibitor bound M^pro^, although these residues showed less binding energies in ABBV-744, Onalespib and Daunorubicin bound M^pro^ complexes. Thus, this assessment discloses that similar binding residues contributing into the overall binding energies of the ABBV-744-M^pro^ complex indicative of ABBV-744 compound binds significantly to M^pro^ enzyme.

## Conclusions

The necessity to control alarming COVID-19 pandemic made us to rationalize potential lead compounds that could be considered in clinical trials. Despite major investigations in the design and development of specific drugs or vaccines, not much proven to be effective against COVID-19. This challenge motivated us to explore the drug designing approaches that could serve informative to combat this disease. In this report, we have performed 3D structure-based pharmacophore modeling followed by virtual screening-based 3D-pharmacophore hypotheses of 75 compounds as potential antiviral agents retrieved from PubChem. Molecular docking workflow using HTVS, SP and XP protocols were used to generate the best hits and their corresponding docked poses. The Six best compounds generated based on their lowest docking binding affinities using XP were considered for ADMET prediction-based physicochemical and pharmacokinetic descriptors and MD simulations analysis. MD simulations approach revealed the two highly selective compounds namely, ABBV-744 and Onalespib possessed significant binding affinity and presumably inhibition of SARS-CoV-2 M^pro^ enzyme. Based on our overall observations, compounds ABBV-744 and Onalespib could be recommended as potential lead for the therapeutic of COVID-19 patients.

## Supplementary information

Supplementary Information 1.

## Data Availability

The data used/generated to support the findings of this study are available from the corresponding author on reasonable request.
