# Supplementary material for: ABBV-744 as a potential inhibitor of SARS-CoV-2 main protease enzyme against COVID-19
Source: Sci Rep. 2021 Jan 8;11:234. doi: 10.1038/s41598-020-79918-3 (PMC7794216; doi:10.1038/s41598-020-79918-3)
Supplement: Supplementary file 1 — Supplementary Information 1. [file 41598_2020_79918_MOESM1_ESM.pdf]

Supplementary Information

ABBV-744 as a Potential Inhibitor of SARS-CoV-2 main Protease Enzyme Against COVID-19

Zeynab Fakhar[1,†] Shama Khan[2,†], Suliman Y AlOmar[3], Afrah Alkhuriji[4] and Aijaz Ahmad[2,5*]

[1] *Molecular Sciences Institute, School of Chemistry, University of the Witwatersrand, PO WITS 2050, Johannesburg, South Africa.*

[2] *Department of Clinical Microbiology and Infectious Diseases, School of Pathology, University of the Witwatersrand, Johannesburg 2193, South Africa.*

[3] *Doping Research Chair, Department of Zoology, College of Science, King Saud University, Riyadh-11451, Kingdom of Saudi Arabia*

[4] *Department of Zoology, College of Science, King Saud University, P.O. Box 2455, Riyadh 11451, Saudi Arabia*

[5] *Infection Control, Charlotte Maxeke Johannesburg Academic Hospital, National Health Laboratory Service, Johannesburg 2193, South Africa.*

† These authors have contributed equally to this work.

*Corresponding Author:

Aijaz Ahmad, Ph.D.

E-mail: Aijaz.Ahmad@wits.ac.za; Aijaz.Ahmad@nhls.ac.za

ORCID: https://orcid.org/0000-0003-2845-0727

Table S1. The considered compounds for the input library.

Entry	Compounds Name	SMILES TEXT
1	AC-55541	c1(c2c(c(=O)[nH]n1)cccc2)[C@H](NC(=O)c1ccccc1)C(=O)N/N=C(/c1cccc(c1)Br)\C
2	ABBV-744	c1(c2c(c(=O)n(c1)C)[nH]c(c2)C(=O)NCC)c1cc(ccc1Oc1c(cc(cc1C)F)C)C(C)(C)O
3	4EGI-1	C1=CC=C(C(=C1)CC(=NNC2=NC(=CS2)C3=CC(=C(C=C3)Cl)Cl)C(=O)O)[N](=O)[O-]
4	4E2RCat	O=C(O)c1cc(c2ccc(/C=C\3/SC(=S)N(Cc4ccccc4)C3=O)o2)ccc1Cl
5	4E1RCat	O=C1/C(=C\c2ccc(c3ccc([N](=O)[O])cc3)o2)/C=C(c2ccccc2)N1c1ccc(C(=O)O)cc1
6	1-Deoxynojirimycin	C1C(C(C(C(N1)CO)O)O)O
7	Zotatifin	c1(c2c(cc(n1)OC)O[C@@]1([C@]2([C@@H]([C@@H]([C@H]1c1ccccc1)CN(C)C)O)O)c1ccc(cc1)C#N)OC
8	XL413	C1CC(NC1)C2=NC3=C(C(=O)N2)OC4=C3C=C(C=C4)Cl
9	Verapamil	CC(C)C(CCCN(C)CCC1=CC(=C(C=C1)OC)OC)(C#N)C2=CC(=C(C=C2)OC)OC
10	Valproic acid	CCCC(CCC)C(=O)O
11	Tomivosertib	c1(=O)n2c(c(cc1Nc1ncnc(c1)N)C)C(=O)NC12CCCCC1
12	Ternatin_4	[C@@H]1(C(=O)N[C@H]([C@H](CC)C)C(=C)N([C@@H](C)C(=O)N([C@@H](CC(C)C)C(=O)N[C@H](C(=O)N([C@H](C(=O)N2CCCC[C@@H]2C(=O)N1)C)C)C[C@@H](C)C=C)C)C)[C@@H](C(C)C)O
13	Siramesine	C1CN(CCC12C3=CC=CC=C3CO2)CCCCC4=CN(C5=CC=CC=C54)C6=CC=C(C=C6)F
14	silmitasertib	C1=CC(=CC(=C1)Cl)NC2=NC3=C(C=CC(=C3)C(=O)O)C4=C2C=CN=C4
15	Sapanisertib	CC(C)N1C2=NC=NC(=C2C(=N1)C3=CC4=C(C=C3)OC(=N4)N)N
16	RVX-208	c1c(cc(c2c1nc([nH]c2=O)c1cc(c(c(c1)C)OCCO)C)O)OC
17	Ruxolitinib	C1CCC(C1)C(CC#N)N2C=C(C=N2)C3=C4C=CNC4=NC=N3
18	RS-PPCC	c1ccccc1[C@@]1(CCN(CC1)C[C@H]1C[C@]1(C(=O)OC)c1ccc(cc1)C)O
19	Rimcazole	CC1CN(CC(N1)C)CCCN2C3=CC=CC=C3C4=CC=CC=C42
20	Ribavirin	C1=NC(=NN1C2C(C(C(O2)CO)O)O)C(=O)N
21	Rapamycin	CC1CCC2CC(C(=CC=CC=CC(CC(C(=O)C(C(C(=CC(C(=O)CC(OC(=O)C3CCCCN3C(=O)C(=O)C1(O2)O)C(C)CC4CCC(C(C4)OC)O)C)C)O)OC)C)C)C)OC
22	Quercetin	C1=CC(=C(C=C1C2=C(C(=O)C3=C(C=C(C=C3O2)O)O)O)O)O
23	PS3061	[C@@H]1(C(=O)N[C@@H](CC(C)C)C(=O)N([C@H](C(=O)N[C@H](C(=O)N([C@H](C(=O)O[C@@H](C(=O)N[C@H](C(=O)N1C)CC(C)C)CCC#N)C)C)CC(C)C)Cc1cn(c2c1cccc2)Cc1ccc(cc1)Br)C)CC(C)C

24	Progestrone	CC(=O)C1CCC2C1(CCC3C2CCC4=CC(=O)CCC34C)C
25	Ponatinib	CC1=C(C=C(C=C1)C(=O)NC2=CC(=C(C=C2)CN3CCN(CC3)C)C(F)(F)F)C#CC4=CN=C5N4N=CC=C5
26	Pioglitazone	CCC1=CN=C(C=C1)CCOC2=CC=C(C=C2)CC3C(=O)NC(=O)S3
27	Pimozide	C1CN(CCC1N2C3=CC=CC=C3NC2=O)CCCC(C4=CC=C(C=C4)F)C5=CC=C(C=C5)F
28	PF-846	c1c2c(ncc1)n(nn2)c1ccc(cc1)C(=O)N([C@H]1CNCCC1)c1c(cccn1)Cl
29	Pepstatin	CC(C)CC(C(CC(=O)O)O)NC(=O)C(C)NC(=O)CC(C(CC(C)C)NC(=O)C(C(C)C)NC(=O)C(C(C)C)NC(=O)CC(C)C)O
30	PD-144418	CCCN1CCC=C(C1)C2=CC(=NO2)C3=CC=C(C=C3)C.C(=O)(C(=O)O)O
31	PB28	c12c(cccc1OC)[C@@H](CCC2)CCCN1CCN(CC1)C1CCCCC1
32	Pazopanib	CC1=C(C=C(C=C1)NC2=NC=CC(=N2)N(C)C3=CC4=NN(C(=C4C=C3)C)C)S(=O)(=O)N.Cl
33	onalespib	CC(C)C1=CC(=C(C=C1O)O)C(=O)N2CC3=C(C2)C=C(C=C3)CN4CCN(CC4)C
34	Olanzapine	CC1=CC2=C(NC3=CC=CC=C3N=C2S1)N4CCN(CC4)C
35	Nitazoxanide	CC(=O)OC1=CC=CC=C1C(=O)NC2=NC=C(S2)[N+](=O)[O-]
36	Nafamostat	C1=CC(=CC=C1C(=O)OC2=CC3=C(C=C2)C=C(C=C3)C(=N)N)N=C(N)N
37	MZ1	N1=C(c2c(n3c([C@@H]1CC(=O)NCCOCCOCCOCC(=O)N[C@H](C(C)(C)C)C(=O)N1C[C@H](C[C@H]1C(=O)NCc1ccc(cc1)c1scnc1C)O)nnc3C)sc(c2C)C)c1ccc(cc1)Cl
38	Mycophenolic acid	CC1=C2COC(=O)C2=C(C(=C1OC)CC=C(C)CCC(=O)O)O
39	ML240	c1c2c(ccc1)nc(n2c1nc(c2c(n1)c(ccc2)OC)NCc1ccccc1)N
40	Minoxidil	C1CCN(CC1)C2=NC(=N)N(C(=C2)N)O
41	Midostaurin	c12c3c(c4c5c1n(c1c2cccc1)[C@@]1([C@@H]([C@@H](C[C@H](n5c2c4cccc2)O1)N(C(=O)c1ccccc1)C)OC)C)C(=O)NC3
42	Metformin	CN(C)C(=N)N=C(N)N
43	merimepodib	COC1=C(C=CC(=C1)NC(=O)NC2=CC=CC(=C2)CNC(=O)OC3CCOC3)C4=CN=CO4
44	Luminespib	CCNC(=O)C1=NOC(=C1C2=CC=C(C=C2)CN3CCOCC3)C4=CC(=C(C=C4O)O)C(C)C
45	Lovastatin	CCC(C)C(=O)OC1CC(C=C2C1C(C(C=C2)C)CCC3CC(CC(=O)O3)O)C
46	Loratadine	CCOC(=O)N1CCC(=C2C3=C(CCC4=C2N=CC=C4)C=C(C=C3)Cl)CC1
47	Lisinopril	C1CC(N(C1)C(=O)C(CCCCN)NC(CCC2=CC=CC=C2)C(=O)O)C(=O)O
48	JQ1	N1=C(c2c(n3c([C@@H]1CC(=O)OC(C)(C)C)nnc3C)sc(c2C)C)c1ccc(cc1)Cl
49	INK-128	c1(c2c(ncn1)n(nc2c1ccc2c(c1)nc(o2)N)C(C)C)N
50	Indomethacin	CC1=C(C2=C(N1C(=O)C3=CC=C(C=C3)Cl)C=CC(=C2)OC)CC(=O)O
51	Ifenprodil	CC(C(C1=CC=C(C=C1)O)O)N2CCC(CC2)CC3=CC=CC=C3
52	Hydroxychloroquine	CCN(CCCC(C)NC1=C2C=CC(=CC2=NC=C1)Cl)CCO
53	Haloperidol	C1CN(CCC1(C2=CC=C(C=C2)Cl)O)CCCC(=O)C3=CC=C(C=C3)F

54	H-89	c1c(c2c(cc1)cncc2)S(=O)(=O)NCCNC/C=C/c1ccc(cc1)Br
55	E-52862	c1ccc2c(c1)cc(cc2)n1nc(cc1C)OCCN1CCOCC1
56	dextromethorphan	CN1CCC23CCCCC2C1CC4=C3C=C(C=C4)OC
57	dBET6	C1(=N[C@@H](c2n(c3c1c(c(s3)C)C)c(nn2)C)CC(=O)NCCCCNC(=O)COc1c2c(ccc1)C(=O)N(C2=O)[C@H]1C(=O)NC(=O)CC1)c1ccc(cc1)Cl
58	DBeQ	c1c2c(ccc1)c(nc(n2)NCc1ccccc1)NCc1ccccc1
59	Daunorubicin	CC1C(C(CC(O1)OC2CC(CC3=C2C(=C4C(=C3O)C(=O)C5=C(C4=O)C(=CC=C5)OC)O)(C(=O)C)O)N)O
60	CPI-0610	c1(ccc(cc1)C1=N[C@H](c2c(c3c1cccc3)c(no2)C)CC(=O)N)Cl
61	compound_10	c1c(cc(c(c1)OC)S(=O)(=O)Nc1ccc(cc1)O)c1sc(nc1C)NC(=O)C(C)(C)C
62	compound_2	c1c2cccc1[C@@H](C)OC(=O)[C@H]1CCCN(N1)C(=O)[C@@H](NC(=O)[C@H](C(C)C)NC(=O)[C@@H]([C@@H](CC/C=C/2)OC)C)C
63	cloperastine	C1CCN(CC1)CCOC(C2=CC=CC=C2)C3=CC=C(C=C3)Cl
64	Clemastine	CC(C1=CC=CC=C1)(C2=CC=C(C=C2)Cl)OCCC3CCCN3C
65	Chloramphenicol	C1=CC(=CC=C1C(C(CO)NC(=O)C(Cl)Cl)O)[N+](=O)[O-]
66	CB5083	c1(ccccc1)CNc1c2c(nc(n1)n1c(cc3c1cccc3C(=O)N)C)CCOC2
67	carbetapentane	CCN(CC)CCOCCOC(=O)C1(CCCC1)C2=CC=CC=C2
68	Captopril	CC(CS)C(=O)N1CCCC1C(=O)O
69	Camostat	CN(C)C(=O)COC(=O)CC1=CC=C(C=C1)OC(=O)C2=CC=C(C=C2)N=C(N)N
70	Birvudine	C1C(C(OC1N2C=C(C(=O)NC2=O)C=CBr)CO)O
71	BD1008	CN(CCC1=CC(=C(C=C1)Cl)Cl)CCN2CCCC2
72	Bafilomycin A1	CC1CC(=CC=CC(C(OC(=O)C(=CC(=CC(C1O)C)C)OC)C(C)C(C(C)C2(CC(C(C(O2)C(C)C)C)O)O)O)OC)C
73	Azithromycin	CCC1C(C(C(N(CC(CC(C(C(C(C(C(=O)O1)C)OC2CC(C(C(O2)C)O)(C)OC)C)OC3C(C(CC(O3)C)N(C)C)O)(C)O)C)C)C)O)(C)O
74	AZ3451	c1c(ccc(c1)C#N)NC(=O)c1ccc2c(c1)nc(n2[C@H](C)C1CCCCC1)c1cc2c(cc1Br)OCO2
75	Apicidin	CCC(C)C1C(=O)N2CCCCC2C(=O)NC(C(=O)NC(C(=O)N1)CC3=CN(C4=CC=CC=C43)OC)CCCCCC(=O)CC

Table S2. The 43 generated compounds using Phase virtual screening.

Entry	Compounds Name	Num Sites Matched	Matched Ligand Sites	PhaseScreenScore
1	4E1RCat	4	A(-) A(3) D(-) D(-) D(5) R(9) R(10)	1.462
2	4E2RCat	4	A(4) A(2) D(-) D(-) D(6) R(9) R(-)	1.376
3	Pimozide	4	A(1) A(-) D(-) D(2) D(-) R(7) R(10)	1.351
4	silmitasertib	4	A(1) A(4) D(-) D(-) D(5) R(9) R(-)	1.321
5	H-89	4	A(2) A(1) D(5) D(-) D(-) R(-) R(9)	1.295
6	Tomivosertib	4	A(3) A(2) D(-) D(-) D(8) R(11) R(-)	1.237
7	PF-846	4	A(1) A(-) D(6) D(-) D(-) R(10) R(12)	1.229
8	4EGI-1	4	A(1) A(3) D(-) D(-) D(5) R(9) R(-)	1.214

9	CB5083	4	A(2) A(-) D(-) D(5) D(7) R(11) R(-)	1.212
10	Azithromycin	4	A(3) A(7) D(16) D(13) D(-) R(-) R(-)	1.179
11	Lisinopril	5	A(3) A(-) D(6) D(8) D(9) R(12) R(-)	1.161
12	Pazopanib	5	A(1) A(3) D(-) D(7) D(6) R(13) R(-)	1.159
13	RVX-208	4	A(1) A(4) D(8) D(-) D(-) R(15) R(-)	1.126
14	AC-55541	4	A(3) A(-) D(8) D(-) D(-) R(13) R(14)	1.092
15	compound_10	5	A(-) A(6) D(9) D(8) D(7) R(12) R(-)	1.073
16	dBET6	4	A(10) A(-) D(13) D(12) D(-) R(22) R(-)	1.068
17	Indomethacin	4	A(3) A(1) D(5) D(-) D(-) R(11) R(-)	1.066
18	Ribavirin	5	A(1) A(6) D(9) D(12) D(10) R(-) R(-)	1.017
19	Zotatifin	4	A(6) A(1) D(-) D(8) D(-) R(15) R(-)	1.014
20	Pioglitazone	4	A(2) A(4) D(-) D(-) D(6) R(10) R(-)	0.999
21	Hydroxychloroquine	4	A(-) A(2) D(-) D(4) D(3) R(10) R(-)	0.998
22	CPI-0610	4	A(1) A(2) D(6) D(-) D(-) R(11) R(-)	0.988
23	merimepodib	4	A(5) A(2) D(-) D(8) D(-) R(-) R(13)	0.934
24	Daunorubicin	5	A(6) A(4) D(13) D(-) D(12) R(-) R(20)	0.911
25	Birvudine	5	A(4) A(3) D(-) D(8) D(7) R(11) R(-)	0.822
26	RS-PPCC	4	A(1) A(2) D(-) D(-) D(4) R(8) R(-)	0.816
27	Chloramphenicol	4	A(1) A(3) D(5) D(-) D(-) R(9) R(-)	0.74
28	Sapanisertib	5	A(1) A(4) D(-) D(6) D(8) R(12) R(-)	0.737
29	INK-128	5	A(3) A(4) D(-) D(8) D(6) R(11) R(-)	0.737
30	Mycophenolic acid	5	A(6) A(3) D(-) D(7) D(8) R(13) R(-)	0.722
31	ML240	5	A(1) A(4) D(5) D(6) D(-) R(10) R(-)	0.692
32	onalespib	4	A(-) A(3) D(4) D(-) D(5) R(10) R(-)	0.687
33	Ifenprodil	5	A(2) A(1) D(-) D(4) D(3) R(-) R(7)	0.674
34	Bafilomycin A1	4	A(1) A(4) D(11) D(-) D(12) R(-) R(-)	0.658
35	Quercetin	6	A(2) A(4) D(10) D(8) D(9) R(15) R(-)	0.56
36	DBeQ	4	A(1) A(-) D(-) D(3) D(4) R(7) R(-)	0.508
37	compound_2	4	A(4) A(6) D(9) D(7) D(-) R(-) R(-)	0.49
38	ABBV-744	5	A(4) A(1) D(7) D(-) D(5) R(13) R(-)	0.488
39	Luminespib	5	A(4) A(5) D(-) D(8) D(7) R(13) R(-)	0.461
40	1-Deoxynojirimycin	5	A(3) A(1) D(8) D(7) D(5) R(-) R(-)	0.444
41	Captopril	4	A(2) A(1) D(-) D(5) D(4) R(-) R(-)	0.419
42	Pepstatin	5	A(7) A(3) D(11) D(16) D(12) R(-) R(-)	0.378
43	MZ1	5	A(7) A(8) D(16) D(-) D(13) R(26) R(-)	0.357

Table S3. Docking-based High Throughput Virtual Screening (HTVS) work flow.

Entry	Compounds name	Glide GScore
1	Pimozide	-7.25

2	RS-PPCC	-6.74
3	4E2RCat	-6.69
4	Daunorubicin	-6.64
5	4E1RCat	-6.24
6	onalespib	-6.23
7	Lisinopril	-6.22
8	ML240	-6.16
9	compound_10	-6.03
10	INK-128	-6.02
11	ABBV-744	-5.97
12	AC-55541	-5.31
13	RVX-208	-5.56
14	RS-PPCC	-5.81
15	dBET6	-5.90
16	Pioglitazone	-5.77
17	CB5083	-5.76
18	PF-846	-5.58
19	DBeQ	-5.52
20	Captopril	-5.50
21	Ribavirin	-5.42
22	Pioglitazone	-5.41
23	Tomivosertib	-5.35

Table S4. Docking-based Standard Precision (SP) Screening work flow.

Entry	Compounds name	Glide GScore
1	ABBV-744	-8.19
2	dBET6	-7.84
3	CB5083	-7.35
4	Pimozide	-7.34
5	AC-55541	-7.28
6	Tomivosertib	-7.31
7	Daunorubicin	-7.20
8	RS-PPCC	-6.67
9	onalespib	-6.90
10	RS-PPCC	-6.52
11	Lisinopril	-6.61
12	Pimozide	-6.49

Table S5. Docking-based Extra Precision (XP) Screening work-flow.

Entry	Compounds name	Glide_XP_GScore
1	**Daunorubicin**	**-9.33**
2	**Onalespib**	**-8.21**
3	**ABBV-744**	**-7.79**
4	dBET6	-7.53
5	RS-PPCC	-6.30

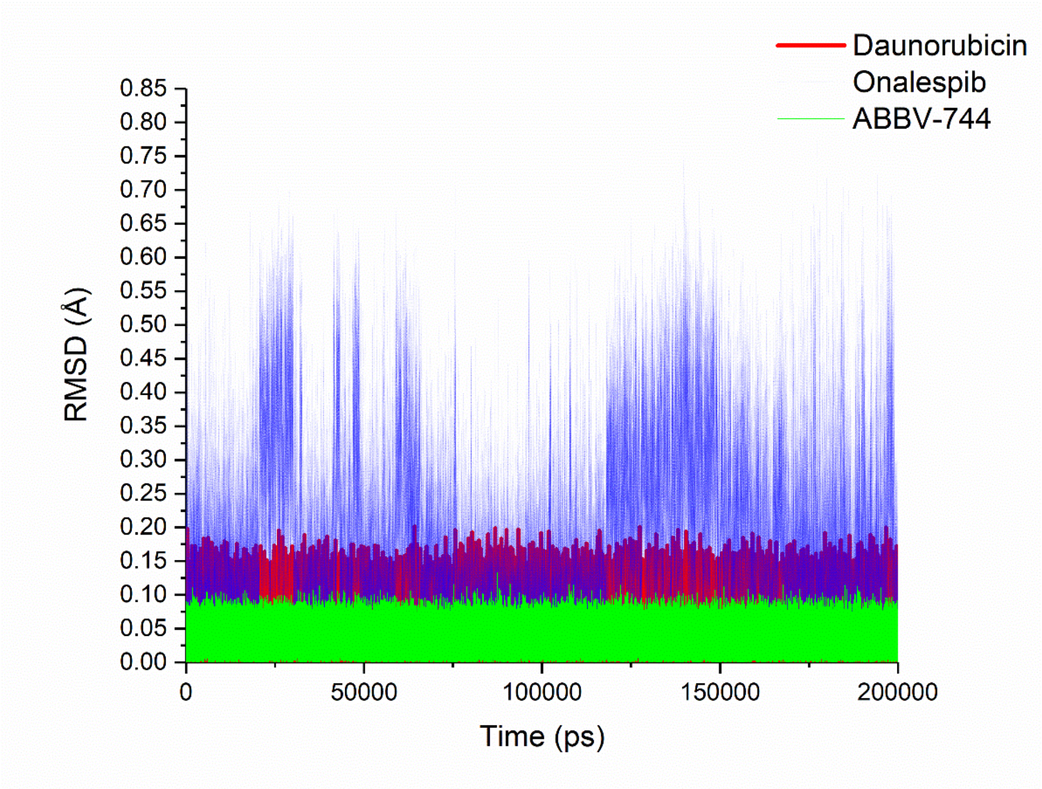

Figure S1. The plot of ligand RMSD of ABBV-744 (green), Onalespid (blue dotted line) and Daunorubicin (red) are presented.
